# The Cancer Screening and Survivorship Program at Roswell Park Comprehensive Cancer Center

**DOI:** 10.1007/s11764-023-01521-y

**Published:** 2024-01-31

**Authors:** Tessa Flores, Christina R. Crabtree-Ide, Kathryn M. Glaser, Mary Reid

**Affiliations:** 1 Department of Medicine, Roswell Park Comprehensive Cancer Center, Elm and Carlton Streets, Buffalo, NY 14263 USA; 2 Department of Cancer Prevention and Population Science, Roswell Park Comprehensive Cancer Center, Elm and Carlton Streets, Buffalo, NY 14263 USA

**Keywords:** Cancer survivorship, Cancer surveillance, Cancer center

## Abstract

**Abstract:**

Roswell Park Comprehensive Cancer Center (Roswell) is the only NCI-designated cancer center in New York State outside of the New York City metropolitan area. The Cancer Screening and Survivorship Program combines cancer screening services with survivorship care in a freestanding centralized clinic with providers also dispersed to see survivors in other clinical areas. The aims of the program are to provide comprehensive, patient-centered care to cancer survivors and their families and caregivers by addressing symptoms, supporting wellness, prevention and quality of life, and engaging community primary care providers in a shared-care model. The clinic is led by an onco-generalist, defined as an internal medicine trained physician serving cancer survivor’s medical issues from all cancer disease sites. Roswell’s Cancer Screening and Survivorship Program growth and development is guided by ongoing research related to patient needs and barriers to care, overall quality of life, health promotion and prevention, as well as education and training to build a more robust cancer survivorship workforce. The cancer center leadership has identified the expansion of cancer survivorship paired with community outreach and engagement, PCP outreach and education, and comprehensive cancer screening services as one of the key strategic areas of growth over the next decade. With the investment in our long-term strategic plan, we expect to continue to grow and serve a broader community of cancer survivors and further our research related to the structure and outcomes of our programmatic activities.

**Implications for Cancer Survivors:**

This program provides robust whole-person care for cancer survivors and provides an example of successful infrastructure for cancer survivorship.

## The institution and catchment area

Roswell Park Comprehensive Cancer Center (Roswell) is the only NCI-designated cancer center in New York State outside of the New York City metropolitan area, located in Buffalo, NY. The Cancer Screening and Survivorship Program combines cancer screening services with survivorship care in a freestanding centralized clinic within a large academic cancer center. Providers are also embedded within two oncology disease site-specific clinics to see survivors in other clinical areas. The clinical program is led by a physician board-certified in internal medicine and pediatrics who serves as an onco-generalist and sees survivors from all cancer disease sites. This is a mixed model with a centralized clinic and disease-site specific clinics when the clinical volumes warrant dedicated disease-site specific care. All clinics use the same EHR (Allscripts).

Roswell’s primary catchment area consists of eight counties that span Western New York (WNY), covering both dense urban centers and remote rural areas. Buffalo is the largest city in the region and is the third poorest city of its size in the USA [1]. Roswell’s catchment area includes 82% who identify as White, 10% African American, 3% Asian, 0.7% Native American, and 5.1% who identify as some other race or two or more races [2]. Our catchment area includes three Appalachian counties with patient populations who experience lower access to healthcare, lower educational attainment, and have high rates of families living below the federal poverty line. In addition, Roswell’s catchment area includes portions of sovereign nation land of the Native American Territories of the Haudenosaunee Confederacy.

## History of the program

The Cancer Screening and Survivorship Program was formed in 2015 under the direction of Mary Reid, Ph.D., MSPH, Chief of Cancer Screening and Survivorship in the Department of Medicine. The goal of the program, driven by literature and existing guidelines (National Comprehensive Cancer Network (NCCN), American Society of Clinical Oncology (ASCO)) is to provide comprehensive, patient-centered care to adult and young adult cancer survivors as well as adult survivors of pediatric cancers. In addition, we aim to improve access to comprehensive cancer screening and surveillance, support for families and caregivers, treatment for ongoing symptoms, support for wellness and quality of life, and serve as the platform to engage the community primary care providers in a shared-care model.

The clinic structure included the recruitment of a medical director to set the clinical protocols, advanced practice providers (APP), nurses specific to the program (2), clinical liaisons (2) for scheduling and patient outreach, and a community outreach coordinator. From the inception, providers delivered specialized survivorship care in a centralized clinic focusing on adult, adolescent and young adult (AYA), and long-term pediatric cancer survivors who are now adults. This program was designed as an umbrella program for multiple cancer types and has since grown to include several dedicated disease-site specific survivorship providers (e.g., breast, gynecologic, and hematologic cancers). Since the inception of the program, the care delivery model has two avenues for patients to receive care in the Survivorship Clinic: patients may receive a one-time consult for a survivorship care plan, full physical examination, and referrals to supportive care services, or they can be transferred from initial oncology clinics to survivorship for whole-person, patient-centered care as well as cancer surveillance, screening, and long-term follow-up. Patients may be (1) internally referred by their primary oncology team or (2) self-referred based appointment requestor referred from community oncology clinics, regardless of where they received treatment. At present, most patients are internally referred, and we see approximately 3000 patients each year. We have implemented plans to modify this model and expand community-based external referrals in the next year. The primary cancer diagnosis of most patients is breast (36.9%), followed by gynecologic (10.3%), and hematologic cancers (9%), with more than 25 primary cancer types seen regularly since the program inception starting in 2017. Most patients were white (78%), 8% percent were Black/African American, 0.5% Native American, 1% Asian, and 12% other, unknown, or multiple races.

When the program began, cancer-specific site leaders who oversee the acute cancer care delivery experience were interviewed to determine who would be eligible for survivorship care and inform workflow, referral patterns, and infrastructure decisions around patient notification and timing. Initially, a template was developed in the Allscripts electronic health record (EHR) for the survivorship care plan based on examples published by the American Society of Clinical Oncology (ASCO). Eventually, we identified a separate system called SurvivorPlan® that pre-populates a survivorship care plan (SCP) with data from tumor registry, our regional health information exchange, pharmacy, and the EHR to streamline the SCP for the survivorship clinical team. At present, we have fully incorporated SurvivorPlan® to generate the survivorship care plan in two formats: one for the patients and one for the primary care provider (PCP).

In September 2016, the clinical program officially started based on a strategic business plan that was developed and approved by the Roswell leadership. This plan assumed the estimated volume of cancer survivors by disease site and stage, with 85% of visits being comprehensive and billed at CPT codes 99204/99214 or 99205/99215. The program was provided with a central clinical space located in the Roswell main campus hospital in downtown Buffalo. With generous support from the Roswell Park Alliance Foundation, funds were used to develop an integrative oncology and wellness program that included a full-time acupuncturist and clinical dietician as well as funding for support groups, and to hire a dedicated nutritionist and acupuncturist. The acupuncturist is now self-supported under a fee-for-service structure. The program offers access to nutritional support, physical therapy and rehabilitation, psychology and social work, support groups, cardiology, clinical genetics, smoking cessation, gastroenterology, pulmonology, integrative medicine, and comprehensive cancer screening for second cancers and long-term surveillance. These services are billed under insurance when possible. The program also provides onco-fertility care, partnering with a community fertility clinic for preservation. These additional programs have also been added: sexual health, intimacy, and sleep health. We have established referral protocols for specialists in speech and hearing, endocrinology, and ophthalmology. Since opening the program, we have seen over 4,000 patients with approximately 12,000 visits over the last 7 years.

## Clinical care delivery

**Fig. 1:**
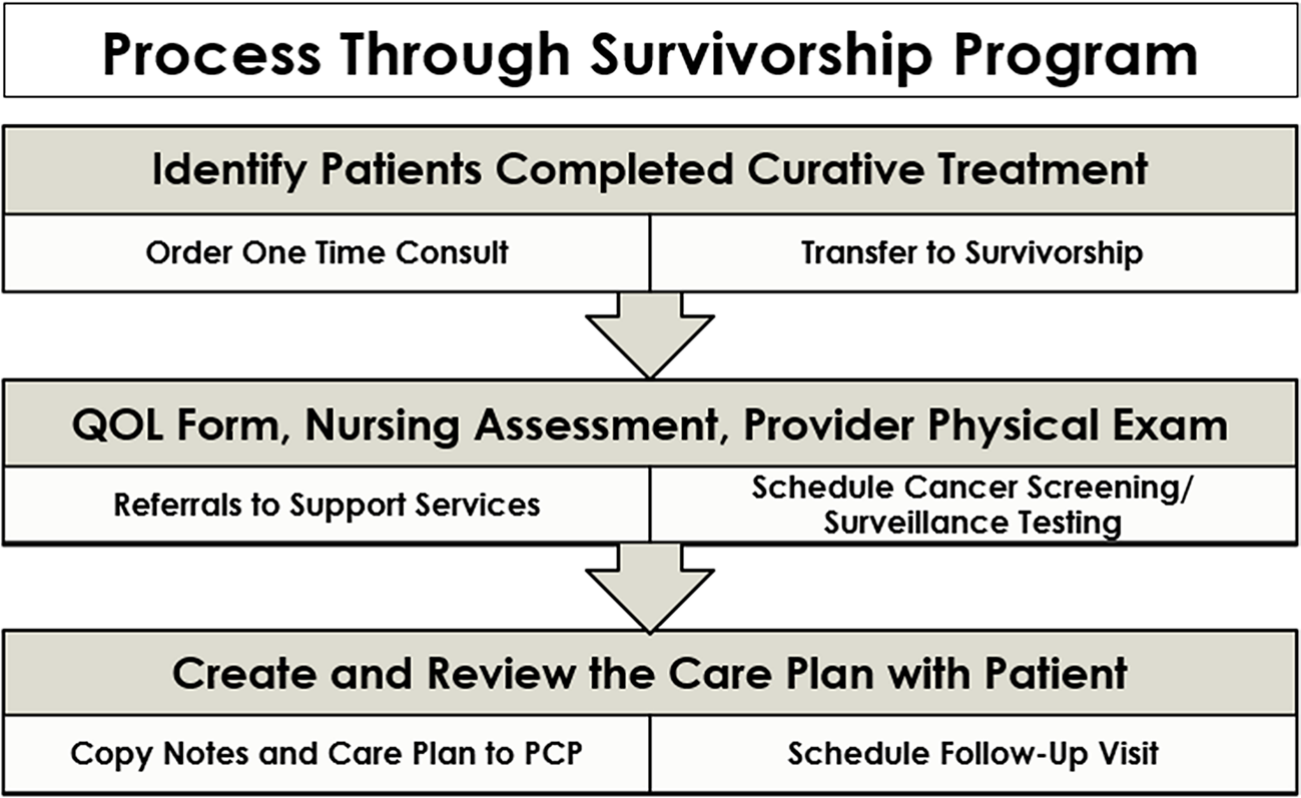
Care model for the survivorship program

In terms of accessing the Survivorship Clinic, patients are referred to survivorship by providers in the acute care oncology clinic, self-referred, or referred by community PCPs. Patients may come for a one-time consultation or transferred from acute care into long-term follow-up and surveillance. Figure 1 shows the steps in accessing the Survivorship Clinic. The clinic appointment includes a complete review of systems and physical exam, quality of life assessment, surveillance imaging and cancer-specific surveillance and blood work, cancer screening for second cancers, and imaging and blood work to monitor for late effects of treatment. We built our clinical structure to reduce testing redundancies such as imaging and a major priority is our system of communicating with the PCPs, both for long-term follow-up needs and for urgent and emergent issues that need to be followed by the patients’ PCPs. To prevent duplicate testing, clinical information from the local health information exchange is reviewed. To foster communication with the patient and their primary care provider (PCP), each new survivor receives a comprehensive survivorship care plan that includes a cancer treatment summary, potential side effects and long-term symptoms, signs of recurrence, health promotion, and prevention recommendations, which is reviewed with the patient at the end of the visit. An abbreviated version of the survivorship care plan is sent to the PCP. The progress note, which is a condensed version of the survivorship care plan, is also sent to the primary care provider to increase awareness of the potential late effects. If urgent medical needs arise that are typically managed by the PCP, the survivorship care provider will immediately call the PCP, facilitating direct communication for management of urgent issue. If the medical need is not urgent, the survivorship team will contact the PCP’s team within days of seeing the patient.

## Clinical team structure

The Medical Director, Tessa Flores, is boarded in pediatrics and internal medicine and oversees the survivorship care for adults. The Adolescent and Young Adult (AYA) Medical Director is Denise Rokitka, a pediatric medical oncologist who oversees the AYA survivorship and onco-fertility program, which is physically embedded in the Survivorship Clinic. The survivorship program clinical structure is an umbrella that covers adult and AYA clinics that are co-located in the same centralized clinic. While Dr. Rokitka is the main pediatric and AYA survivorship provider, and one of the adult survivorship APPs covers oncofertility within AYA survivorship. Currently, there are four full-time APPs (one nurse practitioner and three physician assistants) in the program who specialize in adult, hematologic, and GI care. Two of the APPs practice in the centralized Screening and Survivorship Clinic, and two are embedded in disease site clinics of high volume: hematologic malignancy clinics and gastroenterology oncology clinics. The program also has two registered nurses and two clinical liaisons, who work exclusively in the centralized Survivorship Clinic. The business plan behind the program allows for expansion of staff based on patient volumes; our internal lever is that we begin recruitment when a provider’s template is consistently 85% full.

## Program metrics

Metrics for the comprehensive mixed model survivorship program (all visit types and ages) include (1) visit numbers; (2) visit levels based on time and complexity; (3) numbers of recurrent cancers detected; (4) second primaries diagnosed and downstream revenue; (5) revenue from new to institute patients; (6) downstream revenue from imaging and clinical testing; (7) referrals to support services; and (8) patient satisfaction surveys.

## Clinical service utilization and Commission on Cancer (CoC) guidelines

The primary model for the survivorship care plan development came from materials available from ASCO coupled with the NCCN Survivorship Standards. While CoC did have standards for survivorship care plan completion and delivery to the patient, these standards did not significantly influence the design or conduct of the survivorship program at Roswell. At the time the survivorship program started, this program included a multidisciplinary team of providers and referrals to multiple support services. In 2015, the CoC standards were focused on the completion of survivorship care plans rather than ongoing survivorship care delivery. Despite training and paying acute clinical oncology providers to complete the required number of survivorship care plans, Roswell was not able to meet the initial CoC standard. When the CoC standard was updated (4.8 version) to have a greater focus on care delivery including support services and coordination, Roswell was well-positioned to meet the requirements. We have a multidisciplinary team, we exceed the minimum of three referral support services, and we provide annual seminars for survivors in our community. In 2022, we referred to 508 support services across Roswell, and since program inception we have captured 248 recurrent cancers and 520 cancers The clinical team continues to see great value in the SCPs as a tool for educating patients (adult, AYA, and pediatric) and community providers and therefore they are provided to every patient seen through the program.

## Research and education program components

Our program hosts a monthly Survivorship Translational Research Group (TRG), which consists of 54 members and includes clinical and research faculty from disciplines across the cancer center. There are eight faculty members who conduct research, largely related to symptom management, health promotion, prevention, and building and testing models of cancer survivorship in the community setting. To date, publications and presentations from this group have predominantly focused on describing the features of the program, and we present about our survivorship program to community and/or academic partners at least monthly [3, 4]. The program conducted two needs assessments of survivors using a validated instrument to identify new programmatic needs, one in 2015 to identify needs as the program was being built. This survey was mailed to 35,420 active patients and 1,054 responded. A second needs assessment was disseminated in 2019 to evaluate patient-reported needs for new and existing programs. In 2019, we sent a survey link to 37,020 cancer survivors who received care at Roswell and received 1,690 responses. The surveys included demographic information, cancer history, treatment received, comorbidities, lifestyle choices, healthcare utilization, financial toxicity, symptom management/supportive care, wellness, and integrative medicine services. We used these results to drive program infrastructure decisions. For example, we added nutrition classes, more wellness activities, and continued to expand yoga, all based on patient-reported preferences.

A survey of primary care providers in the WNY communities will be launched in early 2024 to assess the working knowledge of survivorship. A consolidated research database of clinical and patient-reported outcomes from the EHR is under construction. These data include treatment summaries, patient reported symptoms, psychosocial and mental health comorbidities, QoL assessments, referral patterns, and medication use.

In addition, the Survivorship Clinic recruits patient populations for pilot studies and interventional research in areas such as assessments of health literacy, financial toxicity, depression, and physical activity. For example, the identification of survivors with complaints of sleep disturbance on the QoL questionnaire provided pilot data for a successful R01 award focusing on the implementation of nurse-initiated sleep health instruction for cancer survivors [5]. Patients seen in the Survivorship Clinic with documented distress after curative treatment for breast, colorectal or prostate cancers were recruited to standard care or a remote problem-solving skills training (PSST) intervention. This study showed that PSST can improve mood and self-management in cancer survivors [6]. These research activities have been funded by the NCI and the Roswell Park Alliance Foundation.

We provide survivorship education for APPs, internal medicine, medicine, and pediatric residents. Trainees are educated about survivorship care through rotations or shadowing. We have successfully implemented a fellowship in Survivorship Nurse Practitioner (NP) training program with Columbia University and Basset Health Care Network, located in Central New York. In addition, Roswell Survivorship TRG members co-lead the New York State Department of Health Cancer Consortium Survivorship Action Team (SAT). The SAT has received funding through a Centers for Disease Control Comprehensive Cancer Control grant to develop and implement a hub-and-spoke educational series (Project ECHO®) focused on cancer survivorship care in the state. The aim of the project is to engage with and learn from providers who care for patients living in rural and remote regions.

**Fig. 2:**
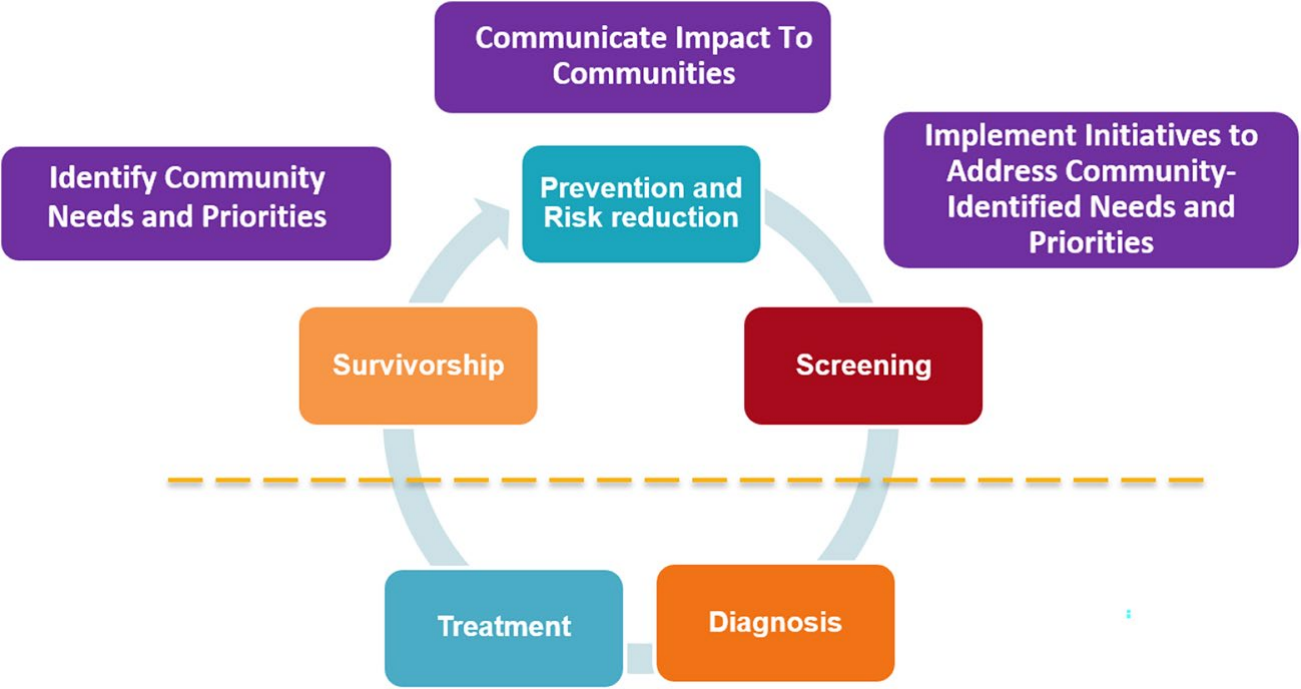
Integrated model of survivorship, screening, prevention and risk reduction, and community outreach and engagement. Items above the dotted line are addressed in the Cancer Screening and Survivorship Program. Data in purple mean community outreach and engagement support

A community outreach and education program was developed, consisting of teams traveling to PCP offices and clinics to gather information about patient-facing barriers to care and providing educational seminars about cancer survivorship, long-term effects of cancer care, symptom management, and follow-up care. From these quarterly meetings with local PCPs, we adjust workflows and communication strategies based on feedback from community providers. Finally, Roswell supports educational and social events on cancer survivorship in our rural outlying regions for patients and caregivers, and a large annual survivorship workshop (Chapter 2) for patients and caregivers in the Buffalo metropolitan region. Figure 2 shows the components of the survivorship program, including our integration with the community outreach and engagement team.

## Challenges, successes, opportunities, and future plans

A major challenge we face is the steady pressure on oncologists to increase productivity seeing more new patients in addition to patients receiving active cancer treatment. One newly diagnosed patient appointment is created for every four survivors taken off an oncologist’s schedule, calculated based on our internal visit templates. Despite this, significant numbers of appointments for both medical and surgical oncology providers continue to be occupied by long-term cancer survivors. Automatic referrals for consultation or messages suggesting a transfer to survivorship in the EHR are needed to promote the flow of patients from busy oncology clinics to the survivorship program, and we are advocating for these solutions. Continued education of providers and patients will also support more patients being transitioned to patient-centered care provided in the Survivorship Clinic.

The survivorship program at Roswell has had several important successes. First, Roswell leadership has identified the integrated expansion of cancer survivorship, comprehensive cancer screening, and community outreach and engagement (COE) as one of the strategic areas of growth for the next decade (Fig. 2). This effort includes a substantial investment in additional personnel and resources to replicate and tailor our program within a spectrum of communities with wide variation in infrastructure and populations experiencing high cancer burden. For example, certain sections of Buffalo are racially and ethnically diverse including populations with known cancer disparities, substantial financial needs, but also many community-based organizations and resources to support. Additionally, our catchment area also includes rural communities with severe poverty rates, few medical facilities, and limited community support systems. To expand services and support to communities with different needs and resources, the COE will engage with the local community stakeholders and community members, consolidate and examine available cancer detection rates, existing resources, and tailor the program based on community feedback and cancer burden. As part of the expansion, our survivorship clinical program will be based in the community, making it easier for patients to access these services, regardless of whether they were treated at Roswell or their geography. This expansion offers an opportunity to standardize and improve survivorship care across the Western and Central regions of New York State. We have started to have an impact on the broader community with regular outreach and education programs and by information gathered about patient-facing barriers during the program’s quarterly outreach visits to PCP offices.

Second, we have had the opportunity to present Roswell’s survivorship program and approach to survivorship care at the Annual Cancer and Primary Care Research International Network (Ca-PRI) in Oxford England in 2023 and at the National Academy of Sciences National Cancer Policy Forum on *Developing a Multidisciplinary and Multispecialty Workforce for Patients with Cancer, From Diagnosis to Survivorship: A Workshop* in 2023.

The Roswell Survivorship Program still cares for only a modest fraction of the patients eligible for survivorship care, despite the support of leadership at all levels. Not only do we provide exceptional patient-centered care with access to a broad array of support services and wellness activities, but we also serve to decompress the oncology services of long-term survivors, opening appointments for active cancer patients. Roswell’s Survivorship Program includes a novel approach to partnering with community outreach and engagement for PCP education, an expanding research program, and an investment into the long-term strategic expansion plan. Through these strengths, we expect to continue to grow and serve a broader community of cancer survivors and further our research related to the structure and outcomes of our programmatic activities. We also anticipate this model of survivorship care in oncology settings will be reproducible both nationally and internationally.
